# Forecasting the Maturation of Electronic Health Record Functions Among US Hospitals: Retrospective Analysis and Predictive Model

**DOI:** 10.2196/10458

**Published:** 2018-08-07

**Authors:** Hadi Kharrazi, Claudia P Gonzalez, Kevin B Lowe, Timothy R Huerta, Eric W Ford

**Affiliations:** ^1^ Center for Population Health IT Department of Health Policy and Management Johns Hopkins Bloomberg School of Public Health Baltimore, MD United States; ^2^ Strategic Management Program Foster School of Business University of Washington Seattle, WA United States; ^3^ The University of Sydney Business School Sydney Australia; ^4^ Department of Family Medicine College of Medicine The Ohio State University Columbus, OH United States; ^5^ Department of Biomedical Informatics College of Medicine The Ohio State University Columbus, OH United States; ^6^ Department of Health Care Organization and Policy School of Public Health University of Alabama Birmingham Birmingham, AL United States

**Keywords:** electronic health records, United States, hospitals, HIMSS EMRAM, Bass diffusion model

## Abstract

**Background:**

The Meaningful Use (MU) program has promoted electronic health record adoption among US hospitals. Studies have shown that electronic health record adoption has been slower than desired in certain types of hospitals; but generally, the overall adoption rate has increased among hospitals. However, these studies have neither evaluated the adoption of advanced functionalities of electronic health records (beyond MU) nor forecasted electronic health record maturation over an extended period in a holistic fashion. Additional research is needed to prospectively assess US hospitals’ electronic health record technology adoption and advancement patterns.

**Objective:**

This study forecasts the maturation of electronic health record functionality adoption among US hospitals through 2035.

**Methods:**

The Healthcare Information and Management Systems Society (HIMSS) Analytics’ Electronic Medical Record Adoption Model (EMRAM) dataset was used to track historic uptakes of various electronic health record functionalities considered critical to improving health care quality and efficiency in hospitals. The Bass model was used to predict the technological diffusion rates for repeated electronic health record adoptions where upgrades undergo rapid technological improvements. The forecast used EMRAM data from 2006 to 2014 to estimate adoption levels to the year 2035.

**Results:**

In 2014, over 5400 hospitals completed HIMSS’ annual EMRAM survey (86%+ of total US hospitals). In 2006, the majority of the US hospitals were in EMRAM Stages 0, 1, and 2. By 2014, most hospitals had achieved Stages 3, 4, and 5. The overall technology diffusion model (ie, the Bass model) reached an adjusted R-squared of .91. The final forecast depicted differing trends for each of the EMRAM stages. In 2006, the first year of observation, peaks of Stages 0 and 1 were shown as electronic health record adoption predates HIMSS’ EMRAM. By 2007, Stage 2 reached its peak. Stage 3 reached its full height by 2011, while Stage 4 peaked by 2014. The first three stages created a graph that exhibits the expected “S-curve” for technology diffusion, with inflection point being the peak diffusion rate. This forecast indicates that Stage 5 should peak by 2019 and Stage 6 by 2026. Although this forecast extends to the year 2035, no peak was readily observed for Stage 7. Overall, most hospitals will achieve Stages 5, 6, or 7 of EMRAM by 2020; however, a considerable number of hospitals will not achieve Stage 7 by 2035.

**Conclusions:**

We forecasted the adoption of electronic health record capabilities from a paper-based environment (Stage 0) to an environment where only electronic information is used to document and direct care delivery (Stage 7). According to our forecasts, the majority of hospitals will not reach Stage 7 until 2035, absent major policy changes or leaps in technological capabilities. These results indicate that US hospitals are decades away from fully implementing sophisticated decision support applications and interoperability functionalities in electronic health records as defined by EMRAM’s Stage 7.

## Introduction

### Background

Technology policy in health care has profoundly affected service delivery and operational efficiencies [1,2]. The period from 2006 to 2016 saw a dramatic increase in electronic health record (EHR) adoption as well as expansion of its functionality [3]. These improvements are attributable to dual environmental pressures [4]. On one hand, the US government put policies into place that provided financial benefits to hospitals for adopting EHRs that met certain criteria [5,6]; on the other hand, internal pressures to adopt EHRs were significant as health systems sought to establish a competitive advantage through operational benefits associated with EHRs [7].

Research in the health care field has closely linked EHR technology adoption to business and clinical outcomes [8-10]. As a result, traditional innovation diffusion analysis, when applied to health care, is complicated by the dynamics found as multiple and varied EHR functionalities are introduced over time. These dynamics represent an opportunity to explore alternative conceptual and analytic approaches to examining technology diffusion and policy interactions.

### Adoption of Electronic Health Records Among US Hospitals

The Health Information Technology for Economic and Clinical Health (HITECH) Act [11] was signed into law with the dual aims of accelerating EHR adoption and promoting their “meaningful use” (MU) by US hospitals [12]. HITECH appropriated billions of dollars to create financial incentives for hospitals that implement EHRs, which meet certain criteria designed to have a meaningful impact on care quality and cost [13]. Hospitals had to attest to MU Stage 2 program eligibility by 2016 to qualify and participate in the reward payments schema [14].

The impact of the HITECH Act has been evaluated in the health services literature so that policymakers can assess the extent to which their intended EHR adoption goals are being realized [15]. Indeed, the HITECH policy resulted in a rapid adoption of EHRs among nonfederal hospitals, increasing the adoption rate from 9.4% in 2008 to 83.8% in 2015 [3]. However, the EHR adoption rates were not equally distributed among all types of hospitals (eg, rural vs urban hospitals) [16], and certain functionalities were adopted earlier than others (eg, MU-mandated functions vs more advanced functions) [17,18].

### Challenges With Using Meaningful Use Data to Assess Electronic Health Record Adoption

In the research literature that focuses on EHR technology adoption, analyses frequently rely on MU data for measuring current use percentages in a binary fashion [3]. In particular, the extant literature on EHR adoption has focused on the transition from paper to electronic data collection or the adoption of a specific function within an EHR [19]. As the adoption of a basic EHR became commonplace, researchers began to frame EHR adoption in terms of its ability to support specific tasks (eg, integrating clinical decision support [CDS] into clinical workflow; automating the collection of patient-reported outcomes; capturing high-quality data for clinical trials; and integrating population health management efforts) [20-26].

Concurrently, hospital planners adopted maturity models that sought to frame EHR implementation as a journey rather than an endpoint. The Healthcare Information and Management Systems Society (HIMSS) Analytics’ Electronic Medical Record Adoption Model (EMRAM) [27] was developed by information technology (IT) and care delivery experts based on the observation that best practices in the industry were path dependent [28,29]. The EMRAM model identifies technological waypoints along an organization’s adaptation journey that are sequential, specific, and measurable [27,28]. For example, closed-loop medical administration requires that decision support software be implemented prior to installing bar code readers that match patients to the prescription drugs they are receiving (ie, need for one level of technology before another level can be adopted as required and measured by the EMRAM model).

### Using Diffusion of Innovation Models to Predict Electronic Health Record Maturation

Diffusion of innovation model produces “technology sophistication forecasts” that predict the degree to which a market or sector has and will adopt sequentially higher levels of functionality in the near future [29,30]. The diffusion of innovation literature and associated methods are critical to understanding and prediction of adoption dynamics. Taken together, these methodological approaches and conceptual frameworks offer a foundation upon which researchers can study the diffusion of innovation in cases where the supporting infrastructure is not replaced, a frequent condition of the Bass model [31]. Furthermore, IT platforms such as EHRs, one where the hardware requirements become secondary to the software innovation, represent a new domain for modeling adoption dynamics using common diffusion models [32].

The purpose of this study was to explore when hospitals will achieve critical EHR functionality. HIMSS Analytics’ EMRAM data and Bass diffusion models were used to assess current EHR capability levels and forecast future diffusion of EHR functionality levels.

## Methods

### Overview

In this study, we explored US hospitals’ EHR technology adoption and implementation patterns accounting for functionality and application upgrades. We used the HIMSS EMRAM data to observe the granular change or progression of EHR functionality among hospitals. The same dataset was used to train the Bass diffusion model and then predict the EMRAM score (ie, the level of EHR functionality) for each hospital. The forecasted scores were aggregated across all hospitals within each future year to depict a national picture of EHR functionality improvements until 2035. We assumed no change in future policies that would affect health IT efforts or EHR functionality (eg, no new MU incentives). Similarly, no dramatic advancement in the technology itself is modeled (eg, effective Natural Language Processing or Artificial Intelligence) as such innovations would change the diffusion curves.

### Data Sources

We used the HIMSS Analytics’ EMRAM data since it provides an MU-comparable EHR adoption measure that takes a more granular approach to assessing functionality uptake (Table 1) [27]. EMRAM data are collected annually across all participating hospitals and are made publically available to interested researchers. HIMSS promulgates its “Annual Study,” which is designed to capture a realistic portrait of the hospital’s IT landscape. The data are submitted via a Web-based portal, phone, or spreadsheets [27]. Given the benchmarking value of these reports, a growing number of hospitals have participated in EMRAM’s Annual Study since 2006. In 2014, over 5402 hospitals (86% of total US hospitals) completed the Annual Study. See Multimedia Appendix 1 for additional details about the EMRAM model and its stages of EHR maturation.

### Theoretical Justification

Modeling the EHR diffusion using an adaptation approach requires two components. First, the technology must track progression through the diffusion stages as set by the “Diffusion of Innovation” theorem, resulting in an “S-curve” to measure the functional form of analysis appropriately [33]. Second, the assumption that new technologies completely displace prior generations needs to be relaxed [34]. Under these two conditions, the Bass “BB-01” model is an appropriate analytic approach to evaluate the diffusion of EHR as it adheres to these requirements [35]. See Multimedia Appendix 2 for additional details of the theoretical justification of using the Bass model to forecast EHR functionality improvement among hospitals.

### Statistical Analysis

The EMRAM data were used as the basis of BB-01 statistical analyses, with estimates calculated in Microsoft Excel using nonlinear regression estimates. Visual Basic, Solver, and the SAS Model Procedure were also used to train and estimate several parameters used by the Bass model [34,35]. The algorithms and macros are publicly available [36]. See Multimedia Appendix 2 for additional details of algorithms used to train the model and predict EHR functionality adoption rates (ie, aggregated EMRAM scores).

**Table 1:** Summary of Electronic Medical Record Adoption Model (EMRAM) stages.

Stage	Description
Stage 0	The organization has not installed all of the three key ancillary department systems (laboratory, pharmacy, and radiology).
Stage 1	All three major ancillary clinical systems are installed (ie, pharmacy, laboratory, and radiology).
Stage 2	Major ancillary clinical systems feed data to a clinical data repository (CDR) that provides physician access for reviewing all orders and results.
Stage 3	Clinical documentation is implemented and integrated with the CDR for at least one inpatient service in the hospital. The Electronic Medication Administration Record application is implemented. Medical image access from picture archive and communication systems (PACS) is available for access by physicians outside the radiology department.
Stage 4	Computerized Practitioner Order Entry for use by any clinician licensed to create orders is added to the nursing and CDR environment along with the second level of clinical decision support (CDS) capabilities related to evidence-based medicine protocols.
Stage 5	A full complement of radiology PACS systems provides medical images to physicians via an intranet and displaces all film-based images.
Stage 6	Full physician documentation with structured templates and discrete data is implemented for at least one inpatient area. Level 3 of CDS provides guidance for all clinician activities. The closed-loop medication administration with bar-coded unit is fully implemented.
Stage 7	The hospital no longer uses paper charts to deliver and manage patient care and has a mixture of discrete data, document images, and medical images within its EHR environment. Clinical information can be readily shared via standardized electronic transactions with all entities that are authorized to treat the patient or a health information exchange.

## Results

### Study Populations and Base Adoption Rates

**Figure 1:**
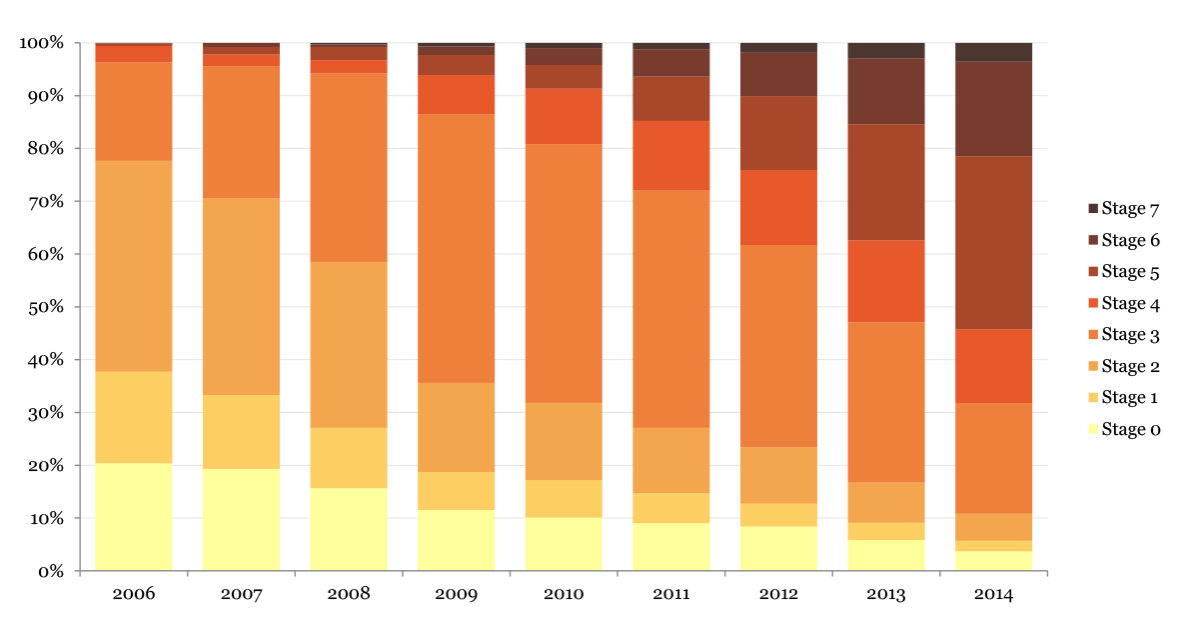
Historical Electronic Medical Record Adoption Model stages among US hospitals from 2006 to 2014.

On average, approximately 5200 hospitals were represented in the EMRAM data across the years studied (2006-2014). The percentage of hospitals achieving various EMRAM stages varied across years (Figure 1). More than 96% of hospitals were identified to be in Stage 3 or below in 2006, while this number decreased to approximately 31% in 2014. Less than 4% of hospitals were in Stage 4 or higher in 2004, while this number dramatically increased across the consequent years: ~6% in 2008, ~20% in 2010, ~38% in 2012, and ~68% in 2014.

### Model Performance

The overall model produced an adjusted R-squared of .91, suggesting a high model fit. Table 2 provides the estimates for the external motivation coefficient (p) and internal motivation coefficient (q) used in the final model (see Multimedia Appendix 2 for additional details). The two motivation coefficients show trends moving in opposite directions. For the earlier stages (ie, EMRAM Stages 1-3), the external influence is the primary motivation for EHR adoption. Starting with Stage 3, the internal influence metric begins to play a more impactful role, and eventually, it becomes the more important factor for EHR functionality adoption in Stages 4 and 5. Given the small number of hospitals that have achieved Stage 6 or 7, interpretation of the motivation coefficients was not undertaken for these stages.

### Electronic Health Record Maturation Forecast

The forecast used EMRAM data from 2006 to 2014 to estimate adoption levels to the year 2035. Table 3 offers a high-level snapshot of the forecasted EHR functionality progression from 2006 to 2035.

**Figure 2:**
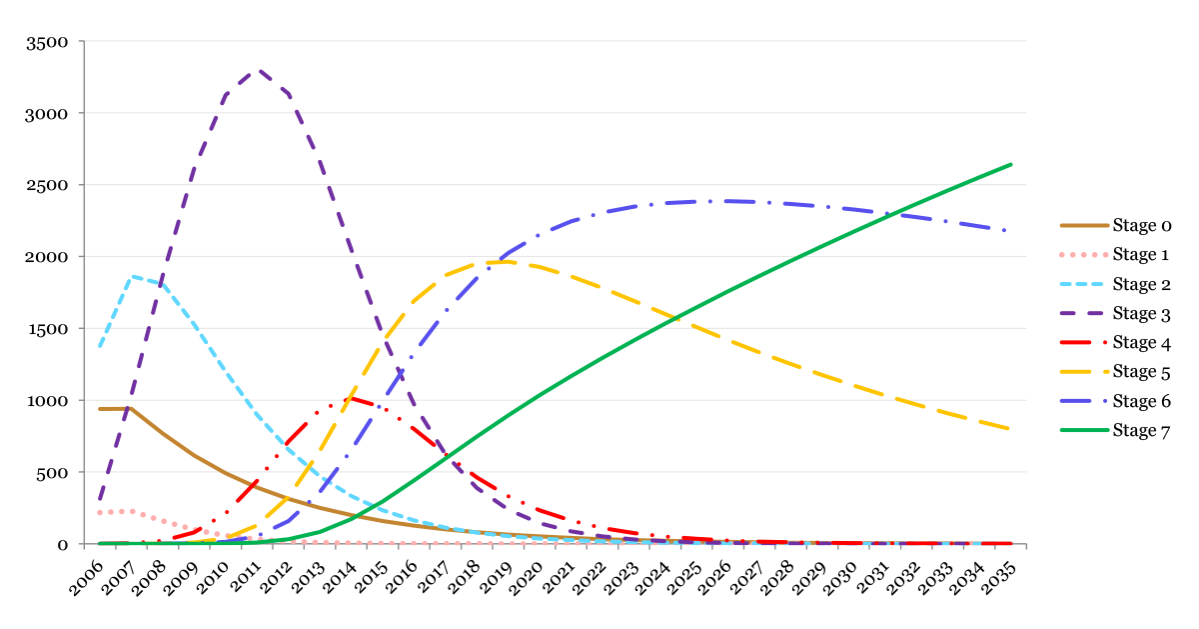
Electronic health record functionality-level adoption among US hospitals using the Electronic Medical Record Adoption Model maturation stages (2014-2035 years are forecasted using the Bass model; vertical-axis represents the number of hospitals).

Figure 1 depicts the forecasted EHR functionality (ie, EMRAM stages) among US hospitals, assuming no major policy or technological changes in the future. Stages 0 and 1 seem to reach their peaks in the first year of observation as the use of EHRs predates 2006 (when HIMSS began to collect adoption data). By 2007, Stage 2 reaches its peak as well. Stage 3 reaches its peak by 2011, while Stage 4 reaches its peak in 2014. The first three stages create a graph that exhibits an “S-curve,” with inflection point being the peak diffusion rate. Assuming current diffusion trajectories, the forecast predicts that Stage 5 will reach its peak by the year 2019 and Stage 6 by the year 2026. Although this forecast extends to the year 2035, no peak was readily observed for Stage 7. A considerable number of hospitals (800+) will stall their EHR adoption at Stage 5, while a higher number of hospitals (2200+) will remain in Stage 6 over an expanded period of time until 2035 (Figure 2).

**Figure 3:**
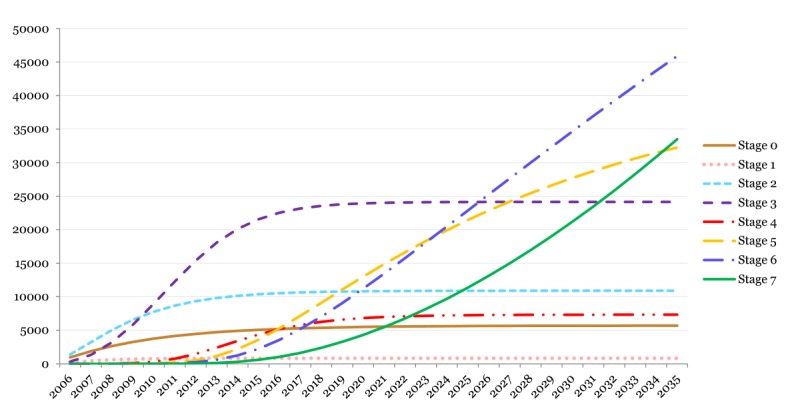
Cumulative electronic health record functionality-level adoption among US hospitals using the Electronic Medical Record Adoption Model maturation stages (2014-2035 years are forecasted using the Bass model; vertical-axis represents the cumulative number of hospitals).

Figure 3 depicts the cumulative volume of hospitals adopting EHRs with higher levels of functionality over the forecasted years. The cumulative volume of hospitals in Stage 4 is constant between the years 2010 and 2014; however, the volume of Stage 5 continues to grow. This is a clear indication of *leapfrogging*, suggesting that adopters either skipped Stage 4 or moved concomitantly with technology adoption for both Stages 4 and 5. Based on the analysis, most hospitals will be focused on the higher stages (Stages 5, 6, and 7) by the year 2025. It is also clear that Stage 7 will not reach a maximum or plateau by the end of the forecast window (Figure 3).

**Table 2:** Parameter estimation and model performance.

Parameter	Stage 0	Stage 1	Stage 2	Stage 3	Stage 4	Stage 5	Stage 6	Stage 7
M^a^	1606	888	2953	100	20	10	5	0
p^b^	0.964	0.234	0.680	0.255	0.015	0.043	0.064	0.026
q^c^	1	1E-9^d^	1E-9	0.120	0.505	0.354	1E-9	0.001

^a^M: market (sample) size for each stage.

^b^p: external motivation coefficient.

^c^q: external motivation coefficient.

^d^1E-9: 0.000 000 001.

**Table 3:** Electronic Health Record (EHR) adoption milestones based on Electronic Medical Record Adoption Model stages.

Rate	Stage 0	Stage 1	Stage 2	Stage 3	Stage 4	Stage 5	Stage 6	Stage 7
50% Year^a^	2008	2006	2008	2010	2014	2021	2025	2027
Max Year	2007	2007	2007	2011	2014	2019	2026	2035^b^

^a^Year that each EHR maturation stage reaches its mid-point.

^b^Stage 7 did not reach a peak in any year until 2035.

## Discussion

### Principal Findings

EHR is a technology platform that allows for the integration of both hardware and software applications designed to improve care quality and increase operational efficiency. To those ends, the United States has introduced policies designed to promote EHRs’ increasingly sophisticated functions. EHRs within hospitals are a prime example of a technology that is adopted and, then, repeatedly updated. This study seeks to outline a direction for future research critical to understanding the dynamics that drive EHR innovation among US hospitals.

We utilized the HIMSS Analytic EMRAM data to assess the EHR functionality levels retrospectively and train the Bass model of diffusion to forecast the adoption of new EHR functionality among US hospitals for the next two decades. The Bass model generated a good explanatory power, and the external and internal influence coefficients mapped closely to the existing regulatory environment. The forecast estimates were also consistent with other literature.

The findings can be evaluated and interpreted in two temporal categories: the retrospective pattern of EHR functionalities identified among the hospitals and the forecasted adoption pattern of EHR functionalities prospectively.

### Retrospective Diffusion Pattern of Electronic Health Record Functionalities (2006-2016)

Given that EHRs had been discussed at the national level for decades before 2006, having Stage-2 as the most prevalent stage in that year is reasonable; however, it is interesting to note that the curve for Stage 1 never exceeded the curve for Stage 0. This phenomenon is the hallmark of leapfrogging and suggests that hospitals moved from Stage 0 directly to Stage 2 or Stage 3 (ie, hospitals adopted multiple generations of functionalities simultaneously rather than adapting them in separate phases). There are two potential explanations for the simultaneous, multistage adoption in the lower EMRAM levels: EHR vendors integrating multiple functions upfront and hospitals being overtly motivated by external factors (eg, MU incentives).

First, EHR vendors may have introduced multiple functions at once. As part of the MU program, the US government introduced an EHR vendor certification regime [37]. Its purpose was to assure hospitals that the EHR platforms would be capable of accommodating future innovations that were likely to be made mandatory features. All of the functionalities and clinical applications delineated in the HIMSS Analytics’ EMRAM Stages 1 through 3 were required components in order for an EHR vendor to be successfully certified [38].

Second, hospitals may have wanted to move through the early stages quickly (Table 2). Considering that the external motivation measures for EMRAM Stages 1 through 3 (ie, p-coefficient: Stage 1 = .234; Stage 2 = .680; and Stage 3 = .255; Table 2) were higher than the internal motivation measures (ie, q-coefficient: Stage 1 = .000; Stage 2 = .000; and Stage 3 = .120; Table 2), the federal government’s MU rewards appeared to have played a significant role in accelerating EHR diffusion. Many hospitals and health systems were incentivized to purchase all or most of the required EHR functionalities from a single vendor rather than having to acquire them separately and in multiple phases [39-41]. This strategy made it possible for hospitals to complete multiple levels at once, allowing them to collect the reward payments in a shorter period [7].

Additionally, hospitals with more recent EHR adoptions may have taken a simultaneous, multistage form in that product vendors began bundling functionalities and clinical capabilities together in a more holistic fashion [41,42]. Similar patterns have occurred in other technologies such as personal computers or mobile phones. Originally, personal computers were sold with little more than an operating system. Consumers had to buy software programs (eg, internet browsers) to be able to use the machine. Later, personal computers came with many preinstalled applications so that consumers could start using their new machines “out-of-the-box.” The net effect is that state-of-the-art information platforms’ minimum feature sets encompass multiple generations of earlier innovations as a technology matures. It is likely that EHR vendors followed a similar pattern of increased technological sophistication as a matter of normal business prior to 2006 [43].

### Prospective Diffusion Pattern of Electronic Health Record Functionalities (2016-2035)

The government’s MU program did not provide rewards or incentives for the later EMRAM stages (ie, Stages 4 through 7). As a result, one of the previously noted major external motivations for adopting higher EHR functionalities was not in play. The BB-01 model effectively controls for this change in motivational factors, suggesting that internal motivation measures play a significantly large role in EHR functionality and clinical application adoption (Table 2). This can be interpreted as hospitals reaching the later EMRAM stages because they are “mission driven” (ie, internally motivated) to adopt the more sophisticated functionalities into their EHR platforms.

The lack of additional EHR incentives in this period will potentially cause internal factors to become the main driver for hospitals to adopt new EHR features. In this scenario, hospitals should observe the imminent need to request and adopt new EHR functionalities to achieve their higher-order goals (eg, quality improvement). For example, EMRAM’s Stage 6 of EHR maturation requires the full adoption of CDS systems across the entire health care system for a variety of clinical practice guidelines. However, if the desired outcomes of a health system, either cost or clinical outcomes, are not aligned with such decision support enhancements in the underlying EHR platform, the hospitals may not have the internal pressure or desire to adopt the new EHR functionalities. Indeed, a complex series of internal factors may disincentivize such progression through EHR functionalities, specifically in a volatile health care market (eg, the cost of aggregating data and embedding a full array of CDS in clinical workflow may outpace the immediate benefits for the hospital). Hence, a considerable number of hospitals are forecasted not to reach Stage 6 by 2035 (Figures 2 and 3). In such a context, EMRAM’s Stage 7 requirements can be harder to achieve as it further pushes the tradeoff between internal factors and expected outcomes by introducing more sophisticated EHR functionalities such as centralized data warehouses that can be readily used for analytical purposes as well as fully interoperable EHRs across hospitals (Table 1; see Multimedia Appendix 1).

Stage 7 of EMRAM requires the development of EHR-derived centralized data warehouses along with extensive analytic infrastructure by hospitals. Although the need for data analytics has grown tremendously among health care providers over the last decade [44], the value of such efforts is not clear for all types of hospitals [17]. On one end of this spectrum, academic medical centers and integrated or value-based delivery systems have realized the need for advanced analytics to push forward with their academic research agenda and quality improvement efforts, hence, accepting or planning for the development of centralized EHR-derived data warehouses. However, on the other end of this spectrum, with fewer internal incentives, smaller critical access and rural or community hospitals may not see the added value of investment in developing complex and often expensive EHR-derived data warehouses, unless the EHR vendors offer it as part of their basic or routine updates without additional charges (eg, EHR vendors attempting to keep their market share). The lack of immediate need for advanced EHR-derived analytics should be further investigated as a potential factor in impeding the attainment of EMRAM’s Stage 7 among underresourced hospitals.

Another major milestone of EMRAM’s Stage 7 for EHR maturation is the interoperability of EHRs among health care providers as well as integration of EHRs with local and regional health information exchanges (Table 1). The challenge of achieving wide interoperability in the health care sector, including hospitals, is a well-known fact, and a variety of causes have been studied (eg, lack of clear guidelines in the MU program) [7,45]. The federal government has extensively persuaded health care providers to adopt interoperability by providing roadmaps and facilitating the development and adoption of new information exchange standards [46]; however, hospital-based EHRs are still largely not interoperable with other settings [45,47,48]. Not reaching Stage 7 of EHR maturity by 2035 is concerning as the continued lack of interoperability may adversely affect patient safety, clinical outcomes, and population health management efforts [48,49]. Future studies should investigate and measure the levels of EHR interoperability among US hospitals and attempt to identify internal and external factors that may impede them from reaching—or drive them to reach—the highest EMRAM score. In addition, hospitals have varying level of capital assets, resources, and IT-driven mindsets that may lead to different adoption patterns of EHR functionalities. Future research should also investigate and discover EHR maturation patterns that are unique to specific hospital groups.

### Limitations

#### Theoretical

Bass model [35] has been used to forecast technology diffusion in a variety of scientific domains [50-52]. The model positions the adoption of technology as either focused on consumers’ replacement of existing products or the adoption of a new technology [34,53]. Furthermore, the more recent Bass “BB-01 Generations” analytic framework, which was used in this study, can be used to model the technological diffusion rates for repeated adoptions where customers upgrade a product as it undergoes rapid technological improvements [35]. However, similar to other simulation studies, the theoretical limits of the Bass model also limit the validity of the results and, consequently, the generalizability of the study [54].

#### Assumptions

We assumed no change in future policies or external factors that may affect EHR functionality advancements or health IT adoption generally (eg, no new MU incentives; stable EHR market for hospital settings) [55]. New health IT policies may change the adoption rate of new EHR functionalities, specifically when incentives are directed for hospitals that are predicted to not achieve the higher stages of EHR maturation [43,56]. Therefore, the findings of this study should be interpreted within the limits of these assumptions and should be updated regularly when new EMRAM data becomes available after the roll out of such policies (eg, Centers for Medicare and Medicaid policies) [57]. Nonetheless, the likelihood of an exogenous factor supporting and reinforcing the adoption of advanced EHR functions is higher than that of a factor demoting the adoption of such functions (eg, more affordable IT infrastructure such as cloud-based EHRs).

#### Data Source

EMRAM does not include EHR adoption data for 2004 and 2005 when health IT policies started to take effect [7]. As this study used data starting in 2006, we could not observe some of the early dynamics that were derived from policies enacted before 2006. Furthermore, this study relies on the definitions and order of stages as defined by HIMSS Analytics in EMRAM (Table 1). Future studies can explore the impact on EHR functionality forecasts if some of these stages were collapsed into fewer categories, if external datasets are merged and used (eg, American Hospital Association’s IT survey) [58], or if new methods are applied to break down the challenges of adopting EHRs into more refined internal or external factors [18].

#### Setting

This study only focuses on inpatient hospital settings and excludes the potential effect of EHR adoption trends in outpatient setting on hospitals. Future studies should investigate the interaction regarding adopting new EHR functionalities between inpatient and outpatient settings [59] (eg, hospitals joining a value-based network may require adopting new EHR functionalities such as higher interoperability with other participating health care providers).

### Conclusion

This study sought to examine when more advanced features of EHRs will be adopted by US hospitals. Using the HIMSS EMRAM data and Bass diffusion models, we were able to forecast the adoption of EHR capabilities from a paper-based environment (Stage 0) to an environment where only electronic information is used to document and direct care delivery (Stage 7). According to the forecast, the majority of hospitals will not reach Stage 7 of EHR maturity by 2035, given that there are no major policy changes.

## Appendix

Multimedia Appendix 1 HIMSS Analytics’ Electronic Medical Record Adoption Model Stages.

Multimedia Appendix 2 Evolution of the diffusion model.

## Abbreviations

CDR: clinical data repository
CDS: clinical decision support
EHR: electronic health record
EMRAM: Electronic Medical Record Adoption Model
HIMSS: Healthcare Information and Management Systems Society
IT: information technology
MU: Meaningful Use
PACS: picture archive and communication systems
