# Supplementary material for: Forecasting the Maturation of Electronic Health Record Functions Among US Hospitals: Retrospective Analysis and Predictive Model
Source: J Med Internet Res. 2018 Aug 7;20(8):e10458. doi: 10.2196/10458 (PMC6104443; doi:10.2196/10458)
Supplement: Multimedia Appendix 1 [file jmir_v20i8e10458_app1.pdf]

Multimedia Appendix 1: HIMSS EMRAM Stages

The EMRAM was created and is administered by HIMSS Analytics and is a framework for evaluating hospitals' information technology implementation (i.e., EHR). EMRAM was developed in 2005 to track EHR functionality diffusion in U.S. hospitals and to recommend a path to complete adoption of optimal EHR applications and functionalities. The EHR capabilities are tracked along a continuum from a paper-based record system (Stage-0) to an environment where only electronic information is used to document and direct care delivery (Stage-7). See the following table for details of the EMRAM stages:

Table A1: Electronic Medical Record Adoption Model (EMRAM) Stages [a]

Stage	Description
Stage 0	The organization has not installed all of the three key ancillary department systems (laboratory, pharmacy, and radiology).
Stage 1	All three major ancillary clinical systems are installed (i.e., pharmacy, laboratory, and radiology).
Stage 2	Major ancillary clinical systems feed data to a clinical data repository (CDR) that provides physician access for reviewing all orders and results. The CDR contains a controlled medical vocabulary, and the clinical decision support/rules engine (CDS) for rudimentary conflict checking. Information from document imaging systems may be linked to the CDR at this stage. The hospital may be health information exchange (HIE) capable at this stage and can share whatever information it has in the CDR with other patient care stakeholders.
Stage 3	Nursing/clinical documentation (e.g. vital signs, flow sheets, nursing notes, eMAR) is required and is implemented and integrated with the CDR for at least one inpatient service in the hospital; care plan charting is scored with extra points. The Electronic Medication Administration Record application (eMAR) is implemented. The first level of clinical decision support is implemented to conduct error checking with order entry (i.e., drug/drug, drug/ food, drug/lab conflict checking normally found in the pharmacy information system). Medical image access from picture archive and

	communication systems (PACS) is available for access by physicians outside the radiology department via the organization's intranet.
Stage 4	Computerized Practitioner Order Entry (CPOE) for use by any clinician licensed to create orders is added to the nursing and CDR environment along with the second level of clinical decision support capabilities related to evidence based medicine protocols. If one inpatient service area has implemented CPOE with physicians entering orders and completed the previous stages, then this stage has been achieved.
Stage 5	A full complement of radiology PACS systems provides medical images to physicians via an intranet and displaces all film-based images. Cardiology PACS and document imaging are scored with extra points.
Stage 6	Full physician documentation with structured templates and discrete data is implemented for at least one inpatient care service area for progress notes, consult notes, discharge summaries or problem list & diagnosis list maintenance. Level three of clinical decision support provides guidance for all clinician activities related to protocols and outcomes in the form of variance and compliance alerts. The closed loop medication administration with bar coded unit dose medications environment is fully implemented. The eMAR and bar coding or other auto identification technology, such as radio frequency identification (RFID), are implemented and integrated with CPOE and pharmacy to maximize point of care patient safety processes for medication administration. The "five rights" of medication administration are verified at the bedside with scanning of the bar code on the unit does medication and the patient ID.
Stage 7	The hospital no longer uses paper charts to deliver and manage patient care and has a mixture of discrete data, document images, and medical images within its EHR environment. Data warehousing is being used to analyze patterns of clinical data to improve quality of care, patient safety, and care delivery efficiency. Clinical information can be readily shared via standardized electronic transactions (i.e., CCD) with all entities that are authorized to treat the patient, or a health information exchange (i.e., other non-associated hospitals, ambulatory clinics, sub-acute environments, employers, payers and patients in a data sharing environment). The hospital demonstrates summary data continuity for all hospital services (e.g., inpatient, outpatient, ED, and with any owned or managed ambulatory clinics). Blood products and human milk are included in the closed-loop medication administration process.

(a) Source: HIMSS Analytic EMRAM documentation[1]

[1] http://www.himssanalytics.org/sites/himssanalytics/files/North_America_EMRAM_Information_2018.pdf (accessed Feb 10 2018)

While each stage reflects the cumulative EHR capabilities of hospitals up to that level, meaning that one is unable to bypass stages, it is possible to adopt the technologies and practices for multiple stages simultaneously. For instance, Stage-1 requires that hospitals have automated information systems for the core ancillary functions (laboratory, pharmacy, and radiology). Stage-2 requires hospitals having an operational “Clinical Data Repository” that contains clinical orders and receives results from the core ancillary departments. However, achieving Stage-2 requires the presence of the automated information systems in Stage-1. In this respect, the EMRAM model identifies technological waypoints along an organization’s adaptation journey that are specific and measurable. As a result, EMRAM scores serve as a way for organizations to understand how close they are to achieving the normative adoption levels.
