# Supplementary material for: Forecasting the Maturation of Electronic Health Record Functions Among US Hospitals: Retrospective Analysis and Predictive Model
Source: J Med Internet Res. 2018 Aug 7;20(8):e10458. doi: 10.2196/10458 (PMC6104443; doi:10.2196/10458)
Supplement: Multimedia Appendix 2 [file jmir_v20i8e10458_app2.pdf]

Multimedia Appendix 2: Evolution of the Diffusion Model

Rogers' technology diffusion model was a first attempt to describe how innovations are accepted or rejected in the marketplace[1]. In particular, the model states that 'innovators' will be the first to adopt and assess new technologies. If innovators are favorable to a new technology, then 'early adopters' are the next group to assess the innovation's utility. If they confirm the innovators' assessment, the new technology may cross the innovation gap (also called the tipping point) to widespread uptake among the 'early majority'. This phenomenon was noted in the healthcare sector with the publication of "Crossing the Quality Chasm: A New Health System for the 21st Century", which recommended EHRs as a critical part of the solution[2]. Subsequent waves are the 'late majority' and, lastly, the 'laggards'.

A core tenet of the diffusion literature states that new technology adoption follows patterns that are predictable and occur in four distinct stages following the classic adoption S-curve[3]. This curve starts with a period of acceleration, which can vary widely depending on the product. The acceleration period's end is marked when the diffusion rates reaches its peak. Acceleration is followed by a corresponding period of deceleration in adoption as the product reaches peak diffusion. Many products then experience a decline in market share as they are replaced by new generations of innovations to complete the life cycle's four stages. It should

---

[1] Greenhalgh T, Robert G, Macfarlane F, Bate P & Kyriakidou O. Diffusion of innovations in service organizations: systematic review and recommendations. *Milbank Quarterly*. 2004;82(4):581-629
[2] Institute of Medicine (US), Committee on Quality of Health Care in America. Crossing the Quality Chasm: A New Health System for the 21st Century. *National Academies Press*. Washington DC. 2001
[3] Rogers EM. Diffusion of Innovations. 5 ed. New York, NY: *Free Press.* 2003

be noted that diffusion and adoption, in this context, are two sides of the same coin. A technology diffuses as it is adopted by actors. As a result, there is a clear unsaid linkage between the two.

While Rogers developed the conceptual model to describe diffusion over time, empirical research by Bass[1] outlined an analytic approach to operationalize the diffusion theory. Within that contribution, Bass identified two latent factors – Internal (p) and External (q) influences – that predict a technology's diffusion pattern, rates of adoption and its drivers. Internal influences are derived from within an adopter's social system and are often referred to as social-contagion. Contagion or social perception[2,3] seeks to explain attitudes, culture and practice through interpersonal interaction. In social contagion, actors are mutually influencing each other resulting in homogeneity within structural subgroups. Bass noted that adoption could be influenced by forces internal to the actor's social relations (e.g. mimetic forces) and sought to incorporate that into Bass' analytic approach. In contrast, external influences, commonly labeled in the diffusion literature as innovation factors, are driven by the technology itself. External influences involve the assessment of the value proposition of the item to the actor. Regulations either promoting or restricting a new technology are common external forces[4].

[1] Bass FM. A new product growth model for consumer durables. *Management Science*. 1969;15:215-227
[2] Karckhardt D. Cognitive social structures. *Social Networks*. 1987; 9(2): 109-134
[3] Freeman LC, Romney AK & Freeman SC. Cognitive structure and informant accuracy. *American Anthropologist*. 1987; 89(2): 310-325
[4] Morrison DG & Raju JS. The marketing department in management science: its history, contributions, and the future. *Management Science*. 2004; 50(4):425-428

The original Bass model was designed to study a single technological iteration at a time; however, many technologies go through rapid evolutions with newer versions replacing older ones. For example, Moore's Law posited that computer memory chips doubled their capacity every year with a concomitant cost reduction[1]. New models have extended the original Bass Model to account for the introduction of new generations of technologies[2]. The newer model was not without limitations. First, the Norton-Bass Model limits the outcome variable to a financial construct – sales of a product that is continuously purchased. Second, the model assumed that "technological substitution proceeds one generation at a time, that is, buyers do not immediately skip the second generation and jump to the third"[3], a phenomenon known as 'Leapfrogging'.

Subsequently, the Bass and Bass algorithm (i.e., BB-01) sought to address these concerns by adjusting for the total count of users at any given level of technology adoption at a specific time[4]. This model incorporates the internal and external influences; however, it also incorporates assessments of leapfrogging. Finally, given that the technology in each generation can be very different, the BB-01 model allows for different treatment of the p and q parameters in each year. The model can use a single p and q for all

[1] Sood A, James GM, Tellis GJ, & Zhu J. Predicting the path of technological innovation: SAW vs. Moore, Bass, Gompertz, and Kryder. *Marketing Science*. 2012;31(6):964-979
[2] Norton JA & Bass FM. A diffusion theory model of adoption and substitution for successive generations of high-technology products. *Management Sciencce*. 1987;33(9):1069-1086
[3] Speece M & Maclachlan DL. Application of a multi-generation diffusion model to milk container technology. *Technological Forecasting and Social Change*. 1995; 49(3): 281-295
[4] Bass PI & Bass FM. IT waves: two completed generational diffusion models. 2004. Retrieved from: http://www.bassbasement.org/F/N/FMB/Pubs/Bass%20and%20Bass%202004.pdf (accessed Feb 20 2018)

generations, a different p and q parameters for each generation, or different parameters for a few generations.

The forecasting model is detailed in equation 1 below, where X_g measures the count of users that will adopt a technology of each generation, g, in a given year, t:

$$X_g(t) = \begin{cases} V_g(t) - V_g(t)F_{g+1}(t), & g < G \\ V_g(t), & g = G \end{cases}, \tag{1}$$

where F_g represents the number of adoptions occurring for each generation, g, in a given period, t, and is defined as:

$$F_g(t) = \begin{cases} \dfrac{1 - e^{-(p_g+q_g)(t-\tau_g+1)}}{\left(1 + \left(\frac{q_g}{p_g}\right) e^{-(p_g+q_g)(t-\tau_g+1)}\right)^2}, & t \geq \tau_g \\ 0, & t < \tau_g \end{cases}, \tag{2}$$

and V_g represents the potential users a technology of each generation, g, in a given year, t:

$$V_g(t) = \begin{cases} M_g(t)F_g(t), & g = 1 \\ M_g(t)F_g(t) + V_{g-1}(t)F_g(t), & 1 < g \leq G \end{cases}, \tag{3}$$

and finally, M_g refers to the market size for each generation. Taken together, these formulae allow interested researchers to calculate the diffusion curves for progressively more sophisticated configurations that are calculated along with the constituent motivation coefficients.
